# Progestins Inhibit Interleukin-1β-Induced Matrix Metalloproteinase 1 and Interleukin 8 Expression via the Glucocorticoid Receptor in Primary Human Amnion Mesenchymal Cells

**DOI:** 10.3389/fphys.2020.00900

**Published:** 2020-07-24

**Authors:** William Marinello, Liping Feng, Terrence K. Allen

**Affiliations:** ^1^Department of Anesthesiology, Duke University Hospital, Durham, NC, United States; ^2^Department of Obstetrics and Gynecology, Duke University Hospital, Durham, NC, United States

**Keywords:** progestins, preterm premature rupture of membranes, glucocorticoid receptor, progesterone receptor, interleukin-1 beta, Interleukin-8, matrix metalloproteinase 1

## Abstract

Preterm premature rupture of membranes is a leading cause of preterm births. Cytokine induced matrix metalloproteinase1 and interleukin 8 production from amnion mesenchymal cells may contribute to fetal membrane weakening and rupture. Progestins inhibit inflammation induced fetal membrane weakening but their effect on the inflammatory response of amnion mesenchymal cells is unknown. This study was designed to determine the role of progesterone receptor membrane component 1 and the glucocorticoid receptor in mediating the effects of progestins on interleukin-1β induced matrix metalloproteinase 1 and interleukin-8 expression in human amnion mesenchymal cells. Primary amnion mesenchymal cells harvested from human fetal membranes were passaged once and treated with vehicle, progesterone or medroxyprogesterone acetate at 10^–6^ M for 1 h followed by stimulation with interleukin-1β at 1 ng/ml for 24 h. Medroxyprogesterone acetate but not progesterone inhibited interleukin-1β-induced interlukin-8 and matrix metalloproteinase 1 mRNA expression. In subsequent dose response studies, medroxyprogesterone acetate, but not progesterone, at doses of 10^–6^–10^–8^ M inhibited interleukin-1β induced interleukin-8 and matrix metalloproteinase 1 mRNA expression. We further demonstrated that inhibition of glucocorticoid receptor expression, but not progesterone receptor membrane component 1 knockdown with small interfering RNA transfection, resulted in a reversal in medroxyprogesterone acetate’s (10^–7^ M) inhibition of interleukin-1β- induced matrix metalloproteinase 1 mRNA expression and interleukin-8 mRNA expression and protein expression. Our findings demonstrate that medroxyprogesterone acetate exerts its anti-inflammatory effect primarily through the glucocorticoid receptor in human amnion mesenchymal cells. Modulation of glucocorticoid receptor signaling pathways maybe a useful therapeutic strategy for preventing inflammation induced fetal membrane weakening leading to preterm premature rupture of membranes.

## Introduction

Preterm birth (PTB) remains a major public health problem in the United States. Despite a slight decline in PTB rates from 2007 to 2014, rates have continued to increase in non-hispanic black women (Martin et al., 2017). Preterm births has multiple etiologies but the leading identifiable cause of preterm birth is preterm premature rupture of membranes (PPROM) (Parry and Strauss, 1998). Preterm premature rupture of membranes contributes significantly to perinatal morbidity and mortality, from adverse effects of prematurity and expectant management, increasing the risks of perinatal infections, placental abruption, umbilical cord prolapse, neonatal respiratory morbidity and adverse neurodevelopmental outcomes (Hadi et al., 1994; Lewis et al., 2007; Lee et al., 2010; Storness-Bliss et al., 2012; Korzeniewski et al., 2014; Ekin et al., 2015). Currently effective strategies for preventing PPROM are lacking.

The pathophysiology of PPROM involves the remodeling in fetal membranes of the extracellular matrix (ECM) in response to inflammation (Kumar et al., 2006). This inflammation induced ECM remodeling ultimately leads to fetal membrane weakening and rupture. *In vitro* biomechanical studies have also demonstrated that the amnion layer is the greatest contributor to the tensile strength of fetal membranes (Moore et al., 2006). The tensile strength of the amnion is due in part to the interstitial collagen type I and III in the compact layer of the amnion secreted by amnion mesenchymal cells in the fibroblast layer (Malak et al., 1993). Amnion mesenchymal cells are also a major source of matrix metalloproteinase 1 (MMP1) which initiates interstitial collagen degradation by cleaving the triple helix of the interstitial collagens (Mogami et al., 2013). Inflammatory cytokines induce MMP1 expression and activity in amnion mesenchymal cells which contributes to collagen degradation in the amnion ultimately leading to fetal membrane weakening and PPROM. Evidence suggesting that MMP1 plays a key role in PPROM include: elevated levels of MMP1 have been detected in the amniotic fluid of PPROM patients in both the presence and absence of infection (Maymon et al., 2000), a single nucleotide polymorphism in the promoter region of the MMP1 gene is associated with an increased risk of PPROM and changes in DNA methylation in the promotor region of the MMP1 gene have been associated with an increased risk of PPROM (Wang et al., 2008).

Our preliminary secretomic analysis of human amnion mesenchymal cells have demonstrated that amnion mesenchymal cells can release interleukin 8 (IL8) in response to interleukin-1 beta (IL1β) stimulation. interleukin 8 is a potent neutrophil chemoattractant and stimulator of neutrophil degranulation. Neutrophils in turn release MMP8 which cleaves the interstitial collagens. Neutrophil infiltration in fetal membranes has been associated with infection induced and abruption induced PPROM (Helmig et al., 2002; Lockwood et al., 2005). IL8 has also been implicated in epithelial to mesenchymal transition – a mechanism which has been implicated in the pathophysiology of PPROM (Radisky, 2005; Janzen et al., 2017). An increase in IL8 levels in amniotic fluid maybe associated with PPROM and predict the onset of preterm labor (Rizzo et al., 1997; Zhang et al., 2000; Jia, 2014). These findings collectively suggest that mesenchymal cells in response to inflammation play a role in the initiation of mechanism that lead to PPROM and PTB.

Progestins are used clinically for the prevention of PTB in women with a prior history of spontaneous PTB (Meis et al., 2003). *In vitro* studies have demonstrated that progestins are able to attenuate inflammation induced fetal membrane weakening (Kumar et al., 2015). The mechanisms by which progestins inhibit fetal membrane weakening still remains unclear. Given the role of the amnion mesenchymal cells in maintaining fetal membrane integrity, the effect of progestins on the inflammatory response of amnion mesenchymal cells may provide some insight into possible progestin-mediated mechanisms. Interestingly, fetal membranes do not express the classical nuclear progesterone receptors but still remain progesterone responsive and this progesterone responsiveness may be mediated through membrane-associated progesterone receptors (Merlino et al., 2009; Luo et al., 2010). For example, fetal membranes express progesterone receptor membrane component 1 (PGRMC1) whose role in fetal membranes remains to be elucidated (Feng et al., 2014, 2016; Allen et al., 2015). Furthermore, in fetal membranes, the amnion expresses higher levels of PGRMC1 when compared with the maternally derived decidual layer (Feng et al., 2014). We have previously demonstrated that PGRMC1 protein expression is diminished in PPROM patients when compared with term and preterm no labor patients highlighting the fact that PGRMC1 may play a role in molecular mechanisms that lead to fetal membrane rupture (Feng et al., 2014). Functionally we have shown that PGRMC1 partially mediates the inhibition of progestins on cytokine induced MMP9 activity in the HTR8 cytotrophoblast cell line and primary amnion epithelial cells (Allen et al., 2014, 2019). PGRMC1 may also play a role in oxidative stress induced senescence in fetal membranes (Feng et al., 2019). These findings demonstrate that PGRMC1 plays a role in maintaining fetal membrane integrity but its role in the amnion mesenchymal cells still remains unknown.

In the absence of the nuclear progesterone receptor, the glucocorticoid receptor (GR) may also explain some of the effects of progestins in fetal membranes. Glucocorticoids have been shown to inhibit lysyl oxidase (LOX) expression via the GR in amnion mesenchymal cells, a mechanism that may lead to fetal membrane rupture *in vivo* (Liu et al., 2016). Another study suggested that the inhibition of inflammation induced fetal membrane weakening *in vitro* by progestins could also be GR mediated (Kumar et al., 2015). Taken together our objectives were firstly to demonstrate that progestins inhibit IL1β-induced MMP1 and IL8 mRNA expression and secondly to determine if this mechanism is mediated through PGRMC1 or GR. Our primary hypothesis was that Progestins inhibit IL1β-induced MMP1 and IL8 mRNA expression primarily through PGRMC1.

## Materials and Methods

### Isolation of Amnion Mesenchymal Cells

The collection of fetal membrane samples was approved by the Duke Medicine Institutional Review Board with a waiver of consent. As a result, fetal membrane samples were deidentified and there was no link to any clinical information. Fetal membrane samples were collected from term healthy patients at elective cesarean section without prior rupture of membranes or labor using a previously described protocol with modifications (Casey and MacDonald, 1996). Briefly, the amnion was separated from the choriodecidua and rinsed three times in Dulbecco modified Eagle medium: Nutrient Mixture F12 (DIMEM/F12) (Thermo Fisher Scientific) media with penicillin, streptomycin and amphotericin-B (Anti/Anti) (Thermo Fisher Scientific). Amnion epithelial cells were released from the amnion which was minced using scalpel blades and then digested with 1 g of 1:250 trypsin in DIMEM/F12 media with Anti/Anti for 30 min at 37°C. The remaining undigested amnion was collected and washed in DIMEM/F12 with Anti/Anti after filtering using a metal strainer. The process was repeated two more times. The undigested tissue fragments from three digestions were then pooled and incubated in DIMEM/F12 with Anti/Anti containing 0.75 mg/ml of Type I collagenase at 37°C for 30 min to release the amnion mesenchymal cells. The isolated cells were collected after filtration of the remaining undigested tissue through a 70 μm cell strainer. The filtrate was centrifuged at 1000 *g* for 5 min and the cell pellet was re-suspended in DMEM/F12 supplemented with 10% fetal bovine serum (FBS) (Thermo Fisher Scientific) and plated in 10 cm culture dishes. The cell cultures were incubated in humidified air and 5% CO2 for 5–7 days until they achieved confluence. Cells were passaged only once using 0.25% trypsin with EDTA and plated at approximately 0.2–0.5 × 10^6^ cells/ml for all subsequent experiments.

### Treatments

Amnion mesenchymal cells were plated at 0.5 × 10^6^ cells/ml for 24 h. To determine the effect of progestin therapy on IL1β-induced MMP1 and IL8 mRNA expression the cell cultures were pre-treated with ethanol, medroxyprogesterone acetate (MPA), or progesterone (P4) (Millipore Sigma) at 10^–6^ M for 1 h followed by stimulation with IL1β at 1 ng/ml (RnD systems) for 24 h in DIMEM/F12 with Anti-Anti and 1% FBS. Subsequent dose response studies were performed using doses of MPA and P4 ranging from 10^–6^ to 10^–8^ M. At the end of the experimental incubation, cell culture media was harvested and centrifuged at 12,000 *g* for 5 min and the supernatant was collected, aliquoted and frozen at −80°C. Trizol^®^ lysates were harvested and frozen at −80°C.

### PGRMC1 and GR Depletion With siRNA

To determine the effect of PGRMC1 and GR on progestin mediated inhibition of IL1β-induced MMP1 and IL8 mRNA levels amnion mesenchymal cells were depleted of PGRMC1 or GR using siRNA. In a separate series of experiments amnion mesenchymal cells were initially plated at 0.2–0.5 × 10^6^ cells/ml for 24 h in DIMEM F12 with 10%FBS. The cultures were then transfected using Lipofectamine RNAiMax and 10 nmol of PGRMC1 siRNA (ID: S21310), GR siRNA (ID: AM513311) or control siRNA (ID: AM4611) for 24 h in both serum and antibiotic free media. After 24 h transfection, the cultures were supplemented with 1 ml of DIMEM/F12 with 20% serum and incubated for an additional 48 h. At the end of the 72 h incubation the cells were then pre-treated with MPA or P4 for 1 h followed by stimulation with or without IL1β 1 ng/ml in DMEM/F12 with Anti/Anti and 1% FBS for an additional 24 h. We assessed the efficacy of PGRMC1 and GR knockdown with siRNA when compared with the control siRNA group using both real-time PCR and Western Blot.

### Quantitation of IL8 and MMP1 Protein Concentrations by Magnetic Luminex Assay

Interleukin-8 and MMP1 levels in cell culture media were quantified simultaneously using the Human Magnetic Luminex assay (RnD systems) as directed by the manufacturer’s protocol. The range of quantitation for MMP1 was 49.8–13,520 pg/ml. The range for quantitation of IL8 was 5.2–1227 pg/ml. Cell culture supernatant samples were diluted 1:10 due to the high concentration of IL8 in these samples to allow measurement within the range of the assay. When IL8 levels were below the lower limit of quantitation, we reported 1/2 of the lower limit of quantitation for IL8. In contrast, MMP1 levels were significantly lower in cell culture media and in the diluted samples they were below the level of quantitation of the assay and were not reported.

### RNA Isolation and Real Time Quantitative PCR

Total RNA was extracted from amnion mesenchymal cells using Trizol, isolated using the RNeasy Mini-Kit and RNA concentrations were quantified using the NanoDrop^®^ spectrophotometer. For each sample, 0.5–1.0 μg of RNA was reversed transcribed into cDNA using the Superscript III^®^ first strand system (Thermo Fisher Scientific). Twenty-five to fifty nanograms of cDNA were used as the template for each real-time PCR reaction. Real-time PCR was performed using pre-validated Taqman probes directed against *MMP1* (assay ID: Hs00899658_m1) and GR (*NR3C1*) (Assay ID:Hs00353740_m1). Forward and reverse primers were used to detect PGRMC1, IL8 mRNA and the housekeeping gene *B2M* mRNA expression (Table 1). We performed Real-Time PCR using the following protocol: initial denaturation at 95°C for 3 min, followed by a 2-step amplification process of 95°C for 30 s and 60°C for 40 s for a total of 40 cycles. Real-Time PCR was performed using the iCycler IQ^TM^ Real-Time PCR detection system (Bio-Rad). All samples were run in duplicates with the mean cycle threshold C_t_ for the gene of interest normalized to the mean C_t_ value for the housekeeping gene *B2M*.

**TABLE 1:** Primer sequences used for real-time quantitative PCR.

**Gene**	**Primer sequence**
IL8	Forward 5′-ACT GAG AGT GAT TGA GAG TGG AC-3′
	Reverse 5′-AAC CCT CTG CAC CCA GTT TTC-3′
PGRMC1	Forward 5′-TGT GAC CAA AGG CCG CAA AT-3′
	Reverse 5′-TGC TTC CTT ATC CAG GCA AAA T-3′
B2M	Forward 5′-GAG GCT ATC CAG CGT ACT CCA-3′
	Reverse 5′-CGG CAG GCA TAC TCA TCT TTT-3′

### Western Blot

At the end of each experiment, cell culture media was removed, and the cells were washed with ice cold PBS and then lysed with radioimmunoprecipitation (RIPA) buffer containing the Complete Mini^®^ protease inhibitor cocktail (Millipore Sigma). Total protein content for each sample was quantified using the Bradford protein assay. An equal amount of protein (10 μg) were separated on a 4–12% Bis –Tris gels (Thermo Fisher Scientific) at 125 V for 60 min and transferred onto a polyvinylidene fluoride (PVDF) membrane. The PVDF membranes were blocked with 5% milk in tris-buffered saline with 0.01% Tween for 1 h and then were incubated with polyclonal rabbit anti-human PGRMC1 (1:2000, catalog No. HPA08277, Millipore Sigma), polyclonal rabbit anti-human GR (1:1000 catalog No. 3660S, Cell Signaling) or monoclonal rabbit anti-human B2M (1:10,000, Catalog No. 12851S, Cell Signaling) antibodies overnight at 4°C. The membranes were then incubated with the appropriate secondary antibody (horseradish peroxidase-linked anti-rabbit or anti-mouse IgG at 1:1000 dilution, Cell Signaling) for 1 h at room temperature, after which they were incubated with the SuperSignal^®^ West Pico Chemiluminescent Substrate (Thermo Fisher Scientific) and exposed on X-ray films. The band densities were quantified using ImageJ^®^ and both PGRMC1 and GR were normalized to B2M.

### Immunofluorescence

Primary amnion mesenchymal cells were plated on chamber slides at 1 × 10^4^ cell/ml in DIMEM/F12 with antibiotics-antimycotics and 10% FBS for 48 h. Ice cold methanol was then used to fix the cells at −20°C for 10 min. The cells were then incubated for 30 min with Image – IT^TM^ Signal Enhancer (Thermo Fisher Scientific) after which they were permeabilized and blocked with 5% goat serum and 0.1% Triton^TM^ X for 1 h. To localize PGRMC1 or GR cells were incubated with rabbit anti-human polyclonal PGRMC1 antibody 1:100 (catalog No. HPA08277, Millipore Sigma) and/or mouse anti-human monoclonal GR antibody 1:250 (catalog no. SAB4800041, Millipore Sigma) overnight at 4°C in a humidified slide chamber. Cells incubated with a monoclonal anti-mouse (catalog no. MA5-14453, Thermo Fisher Scientific) and polyclonal anti-rabbit antibody (catalog no. ab27472, Abcam) were used as negative controls. To determine the homogeneity of the culture the cells were stained with the mesenchymal cell marker mouse anti-human monoclonal vimentin antibody (clone v9, catalog no. M0725, Agilent Dako) at 1:200 dilution. The cells were then incubated for 1 h with the Alexa Fluor^TM^ 488 goat anti-mouse ReadyProbes^TM^ (Thermo Fisher Scientific) and/or Alexa Fluor^TM^ 594 goat anti-rabbit ReadyProbes^TM^ (Thermo Fisher Scientific) diluted based on the manufacturer’s specifications. The cells were then incubated with DAPI 1:1000 for 5 min. ProLong^TM^ Diamond Antifade (Thermo Fisher Scientific) was used as the mounting media and the cells were imaged with the Zeiss Axio Imager fluorescence microscope.

### Statistical Analysis

All experimental groups were compared using one-way analysis of variance (ANOVA) with *post-hoc* pairwise comparisons using the Sidak test with each *P*-value was adjusted for multiple comparisons. A *p* < 0.05 was considered significant. Data were analyzed using GraphPad^®^ Prism. Data are presented as mean ± standard error of the mean (sem).

## Results

**FIGURE 1:**
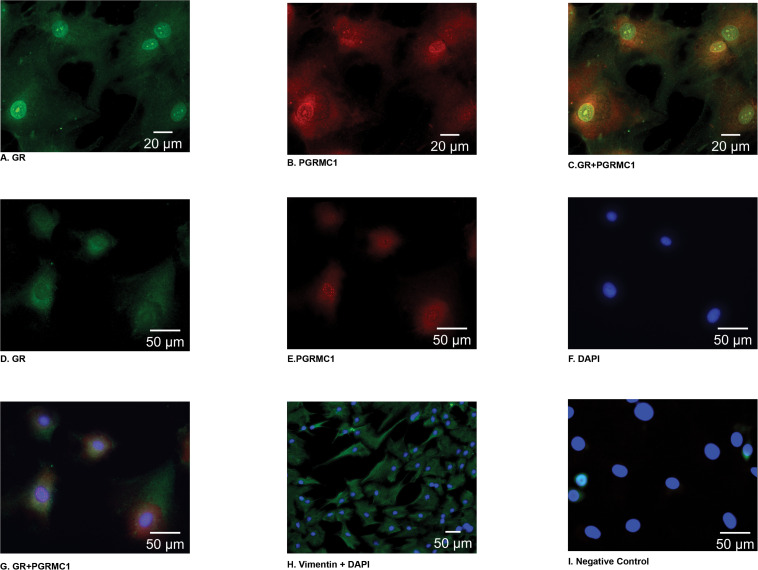
In primary amnion mesenchymal cells GR was localized (green) primarily to the nucleus in some cells **(A)** and the cytoplasm in other cells **(D)**, PGRMC1 (red) localized to the nucleus and the perinuclear area of the cytoplasm **(B,E)**. Colocalization of GR and PGRMC1 in the nucleus when GR is expressed in the nucleus **(C)** vs. when GR is primarily expressed in the cytoplasm **(G)**. Primary mesenchymal cells stained with DAPI **(F)**, vimentin to identify primary mesenchymal cells **(H)**, and the negative control **(I)**. Images **(A–C)** were captured at 63× magnification **(D–G)**, **(I)** 40× magnification, and **(H)** at 20×.

Immunofluorescent staining demonstrated that PGRMC1 is localized to the nucleus, the perinuclear area, and the cytoplasm of amnion mesenchymal cells (Figures 1B,E). Interestingly, GR localized to the nucleus in some cells (Figure 1A) and in the cytoplasm in other cells (Figure 1D). When GR and PGRMC1 were both expressed in the nucleus they appeared to co-localize in the nucleus (Figure 1C). When GR was primarily expressed in the cytoplasm there was no evidence of co-localization with PGRMC1 (Figure 1G). The majority of the cells (>95%) stained positive for the mesenchymal cell marker vimentin (Figure 1H). Primary amnion mesenchymal cells were stained with DAPI to localize the nucleus (Figure 1F). The negative control demonstrated no evidence of non-specific staining (Figure 1I).

### The Effect of Medroxyprogesterone Acetate and Progesterone on IL1β-Induced MMP1 and IL8 mRNA Expression in Amnion Mesenchymal Cells

**FIGURE 2:**
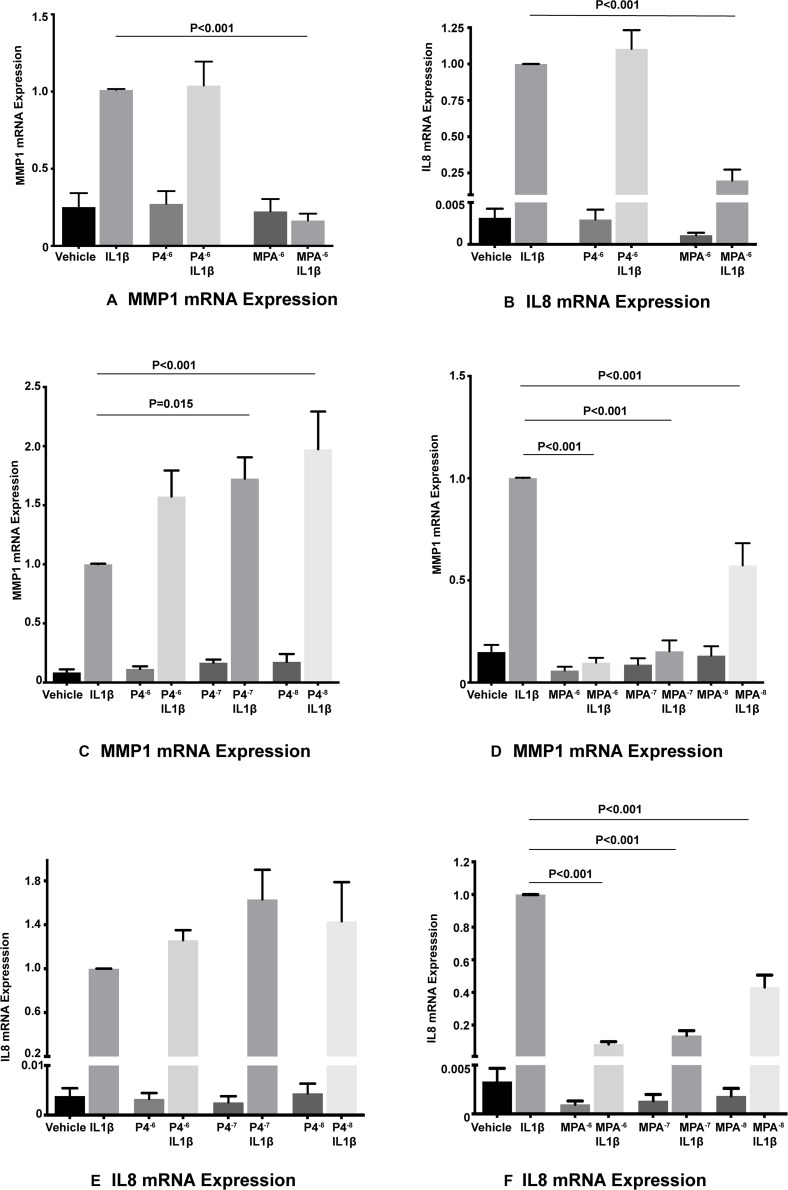
MPA but not P4 inhibits IL1β-induced *MMP1* and *IL8* mRNA expression in primary amnion mesenchymal cells **(A,B)**. P4 dose response studies **(C,E)**, and MPA dose response studies **(D,F)** on IL1β-induced *MMP1* and *IL8* mRNA expression (*n* = 6–7 patients).

Interleukin-1β significantly induced both *MMP1* and *IL8* mRNA levels in primary amnion mesenchymal cells when compared with the unstimulated (vehicle) control. In initial experiments MPA at a dose of 10^–6^ M significantly inhibited IL1β-induced *MMP1* and *IL8* mRNA expression when compared with the stimulated control (vehicle control plus IL1β) while P4 did not show any effects (Figures 2A,B). Both MPA and P4 did not suppress basal *MMP1* or *IL8* mRNA expression in amnion mesenchymal cells when compared with the unstimulated control.

In subsequent dose response studies pre-treatment with MPA at doses of 10^–6^, 10^–7^, and 10^–8^ M significantly inhibited IL1β-induced *MMP1* and *IL8* mRNA expression when compared with the stimulated controls (Figures 2D,F). Surprisingly, pre-treatment with P4 at doses of 10^–7^ and 10^–8^ M were associated with a significant increase in IL1β-induced *MMP1* mRNA expression when compared with the stimulated control (Figure 2C). Pre-treatment with all doses of P4 had no significant effect on IL1β-induced IL8 mRNA expression when compared with the stimulated control (Figure 2E). All doses of MPA and P4 tested had no effect on both basal *MMP1* and *IL8* mRNA expression when compared with the unstimulated control. In the subsequent siRNA experiments, we used MPA at a dose of 10^–7^ M and P4 at a dose of 10^–6^ M.

### The Role of PGRMC1 and GR on Progestins Mediated Inhibition of IL1β-Induced MMP1 and IL8 Expression in Amnion Mesenchymal Cells

**FIGURE 3:**
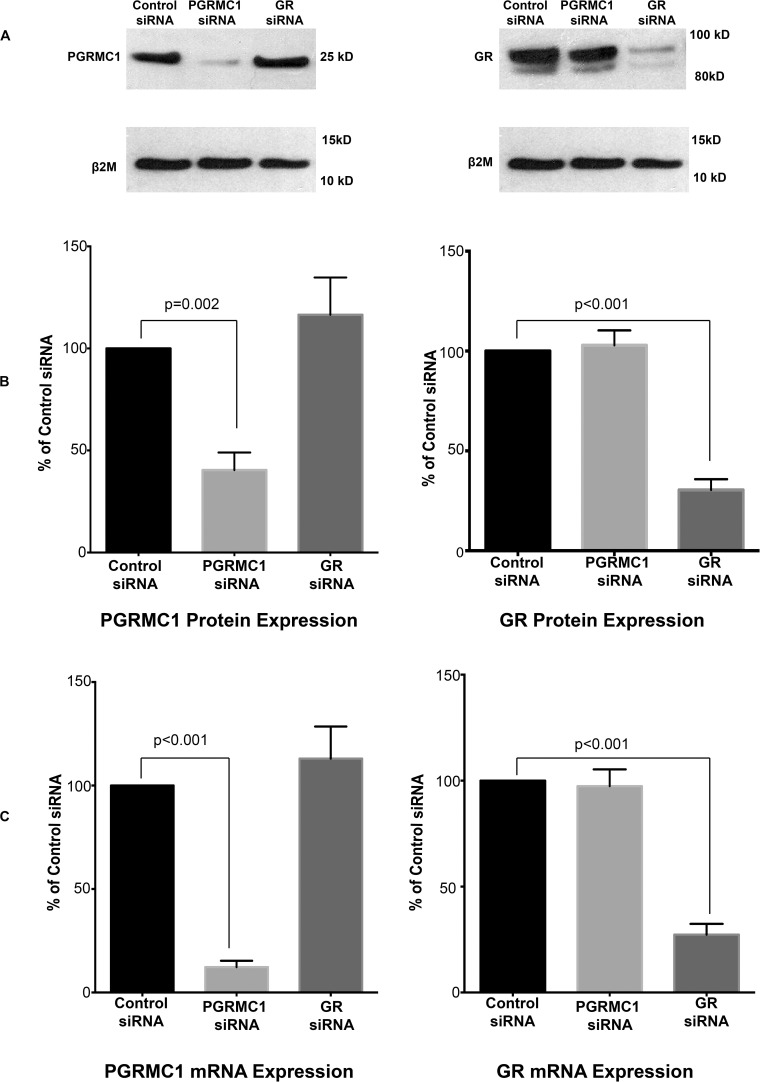
PGRMC1 siRNA significantly inhibits PGRMC1 protein expression **(A,B)** and *PGRMC1* mRNA levels **(C)**. GR siRNA significantly inhibits GR protein expression **(A,B)** and GR mRNA levels **(C)** (*n* = 10 patients). For illustrative purposes the B2M image (top panel lower blot) was reused in both images.

PGRMC1 siRNA significantly inhibited *PGRMC1* mRNA and protein expression in amnion mesenchymal cells but had no significant effect on GR mRNA and protein expression when compared with the control siRNA group (Figure 3). GR siRNA significantly inhibited GR mRNA and protein expression but had no significant effect on *PGRMC1* mRNA and protein expression when compared with the control siRNA group (Figure 3).

**FIGURE 4:**
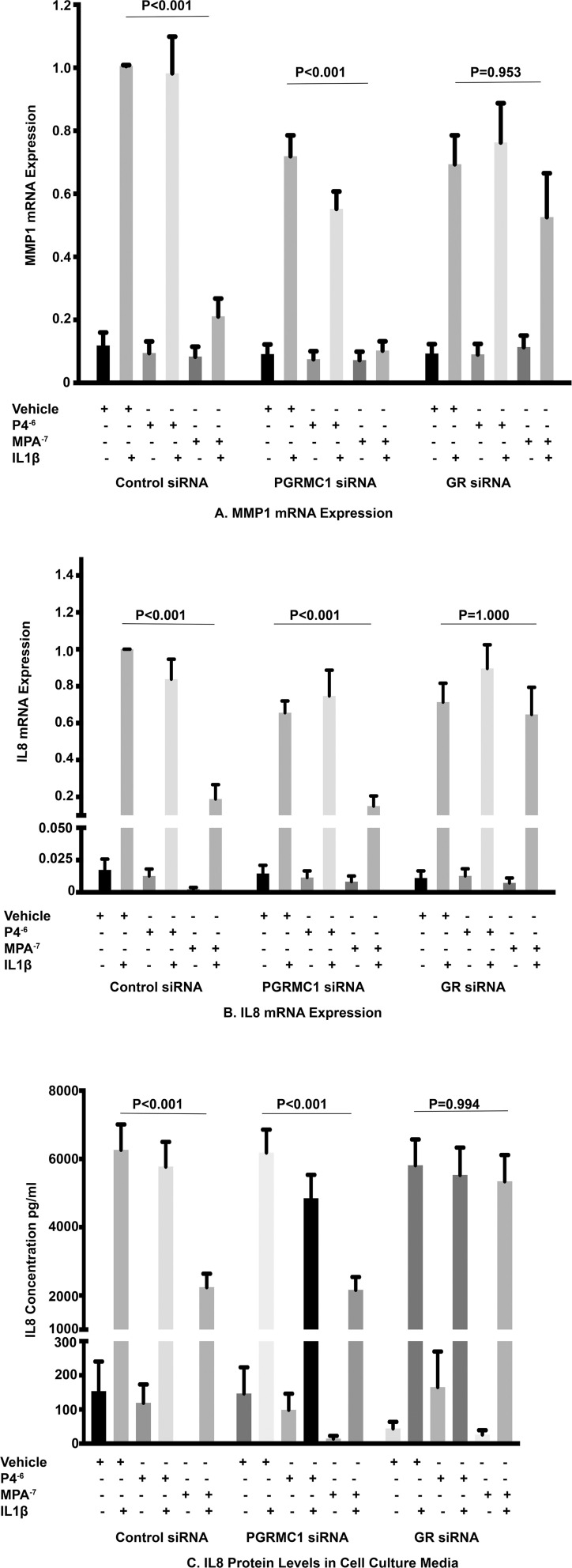
The role of PGRMC1 siRNA and GR siRNA on IL1β-induced *MMP1* mRNA expression **(A)** and IL1β-induced *IL8* mRNA and protein levels **(B,C)** (*n* = 11 patients).

In the control siRNA group, the inhibition of IL1β-induced *MMP1* mRNA expression by MPA at 10^–7^ M when compared with the stimulated control was significantly attenuated by GR siRNA but was unaffected by PGRMC1 siRNA treatment (Figure 4A). As we had previously observed P4 at a dose of 10^–6^ M had no effect on IL1β-induced *MMP1* mRNA expression when compared with the stimulated control in the control siRNA group and this effect was unaffected by PGRMC1 or GR inhibition with siRNA. Furthermore, both MPA and P4 had no significant effect on basal MMP1 mRNA expression and this was unaffected by PGRMC1 and GR inhibition by siRNA.

In the control siRNA group, the inhibition of IL1β-induced *IL8* mRNA expression and IL8 protein levels in cell culture media by MPA at a dose of 10^–7^ M when compared with the stimulated control was significantly attenuated by GR inhibition with siRNA but was again unaffected by PGRMC1 inhibition with siRNA (Figures 4B,C). Progesterone at a dose of 10^–6^ M had no significant effect on both IL1β-induced IL8 mRNA expression and protein concentration when compared with the stimulated control in the control siRNA group and this lack of effect was unaffected by either PGRMC1 or GR inhibition. Both MPA and P4 had no significant effect on basal IL8 mRNA expression or protein concentration when compared with the unstimulated control and this was unaffected by PGRMC1 and GR inhibition by siRNA.

## Discussion

Our findings demonstrate that MPA but not P4 inhibit IL1β-induced MMP1 mRNA expression and IL8 mRNA levels and secreted protein levels through GR and not through PGRMC1 in amnion mesenchymal cells. These findings are similar to our previous work in amnion epithelial cells which demonstrated that the inhibition of cytokine-induced MMP9 activity and mRNA levels in amnion epithelial cells was mediated through GR (Allen et al., 2019). Additionally, PGRMC1 localizes to the perinuclear area and the nucleus which is stark contrast to its expression pattern in amnion epithelial cells where it primarily localized to the cytoplasm and perinuclear area (Allen et al., 2019). GR in turn localizes to both the nucleus and cytoplasm as has been previously described (Wikström et al., 1987). Since in the inactive state, GR is localized to the cytoplasm as a part of a multiprotein complex with chaperone proteins and immunophilins, localization to the nucleus could represent activation by ligands present in cell culture media (Matthews et al., 2011). Alternatively the heterogeneity of GR localization in amnion mesenchymal cells could represent ligand independent trafficking of GR between the nucleus and cytoplasm which may occur normally during different phases of the cell cycle (Matthews et al., 2011). Both proteins appear to co-localize to the nucleus and even though the clinical relevance of this co-localization remains unclear, it could represent a functional interaction between both receptors in regulating genes involved in inflammation, cell cycle regulation and apoptosis in amnion mesenchymal cells and fetal membranes (Peluso et al., 2010; Allen et al., 2014, 2019; Sueldo et al., 2015).

This study provides further evidence that the anti-inflammatory effects of progestins, specifically MPA in the amnion are primarily mediated through GR and this is particularly important in fetally derived cells that lack the nuclear progesterone receptor (Merlino et al., 2009; Allen et al., 2014). The findings are even more important given the central role that amnion mesenchymal cells play in collagen turnover and immunomodulation in fetal membranes (Casey and MacDonald, 1996). The classic glucocorticoid receptor GRα, which is ubiquitously expressed, is known to mediate most of the known biological effects of glucocorticoids. However, there is still sparse data on the expression patterns of GR in fetal membranes in the preterm delivery phenotypes. This is further complicated by the fact that alternative mRNA splicing and translation initiation sites leads to the generation of multiple GR isoforms (Lu and Cidlowski, 2005; Lu et al., 2007; Turner et al., 2007). Currently, at least 8 GR isoforms have been identified in the placenta and are affected by gestational age and fetal sex and the roles of these isoforms still remain unclear (Saif et al., 2015). GRα-A is one of the isoforms involved in mediating glucocorticoid effects through its ability to transcriptionally activate and repress multiple gene targets. Interestingly the relative expression of GRα-A in the nucleus is less in preterm placentae than term placentae (Saif et al., 2015). The GR isoforms in the fetal membranes remain unknown, however, identification of the isoforms which mediate anti-inflammatory effects in fetal membranes could allow the development of safer glucocorticoids with reduced side effects.

Our findings in this study highlight the need for elucidating the underlying mechanism by which GR exerts these effects in fetal membranes. Recently it has been determined that distinct negative glucocorticoid response elements (nGRE) mediate the transcriptional repression effects of GR via an inverted quadrimetric palindrome separated by 0–2 nucleotide pairs (Surjit et al., 2011). Alternatively the protein-protein interaction between GR and specific transcription factors at promoters can result in inhibition (or stimulation) of target genes. These promoters either do not contain GRE (tethering) or have both GREs and responsive elements for the transcription factors that associate with GR (composite promoters) (Reichardt et al., 1998). Transcription factors that have been implicated in this protein-protein interaction include NF-κB, AP-1, and STATs (Heck et al., 1994; Stöcklin et al., 1996; De Bosscher et al., 1997). Interestingly NF-κB, AP-1 and STATs are some of the key transcription factors involved in the transcriptional regulation of MMP1 and IL8 gene expression (Chaudhary and Avioli, 1996; Overall and López-Otín, 2002; Fanjul-Fernández et al., 2010; Lin et al., 2016). Elucidating the anti-inflammatory mechanisms of GR in the amnion mesenchymal cells may allow the development of tissue specific GR modulators which inhibit inflammatory induced fetal membrane weakening and PPROM.

The role of PGRMC1 also remains unclear in fetal membranes. PGRMC1 did not mediate MPA’s anti-inflammatory effect and we were also unable to demonstrate an anti-inflammatory effect of progesterone on IL1β-induced inflammation in amnion mesenchymal cells. However, emerging evidence demonstrates that PGRMC1’s effects maybe cell type specific. In our previous work we demonstrated that PGRMC1 partially mediated the inhibition of TNFα-induced MMP9 activity by MPA in HTR8 cells, a cytotrophoblast cell line and to a lesser extent in primary human amnion epithelial cells (Allen et al., 2014, 2019). More recently we have demonstrated that PGRMC1 plays a role in mediating oxidative stress induced cellular aging through p38 MAPK and SIRT3 in primary human chorion cells (Feng et al., 2019). It has also been demonstrated that PGRMC1 may have anti-inflammatory effects by suppressing TNFα-induced gene expression independent of progesterone in N42 hypothalamic cells (Intlekofer et al., 2019). While in this study we were unable to demonstrate that PGRMC1 plays a role in IL1β-induced inflammation in amnion mesenchymal cells, it is likely that it may regulate other pathophysiological pathways that lead to PPROM and therefore warrants further investigation.

Interestingly in our *in vitro* study P4 was ineffective in preventing IL1β-induced MMP1 and IL8 expression and lower doses of P4 were associated with increased IL1β-induced MMP1 mRNA expression. In our prior work we have also been unable to demonstrate that P4 effectively prevents cytokine induced MMP9 activity and mRNA expression in primary amnion epithelial and chorion cells (Allen et al., 2015). The lack of effect of P4 and its augmentation of IL1β-induced MMP1 mRNA expression at lower doses could partly be explained by the lack of expression of the nuclear progesterone receptor in amnion mesenchymal cells and modulatory effects mediated via GR, respectively. The nuclear progesterone receptor isotypes PR-A and PR-B mediate most of the anti-inflammatory actions of P4 (Patel et al., 2014). In fact PR-A and PR-B null female mice demonstrate marked inflammatory changes in the endometrium (Lydon et al., 1995). Therefore in the absence of the nuclear progesterone receptor, P4’s anti-inflammatory effects maybe significantly attenuated. However, P4 also binds GR but it does so with low relative affinity and it may also act as a weak partial agonist for GR-mediated transactivation and transreprepresion (Fuhrmann et al., 1996; Koubovec et al., 2005; Africander et al., 2011). Therefore the augmentation of IL1β-induced MMP1 mRNA expression could represent dose dependent conformational changes leading to GR-mediated transactivation and expression of proinflammatory genes. These anti and proinflammatory GR mediated effects highlight the complexity of GR signaling and the importance of finding the middle ground in maximizing GR-mediated therapeutic benefits.

Significant controversy surrounds the clinical use of progesterone for PTB prevention. Preclinical studies have demonstrated that progesterone promotes uterine quiescence by suppressing the expression of contraction associated proteins, inhibiting the expression of proinflammatory chemokines and cytokines and inhibiting immune cell infiltration and activation in the myometrium, potentially preventing mechanisms that may lead to PTB (Lei et al., 2015; Nadeem et al., 2016; Edey et al., 2017; Amini et al., 2019). In the cervix functional progesterone withdrawal is also associated with a local increase in proinflammatory mediators, matrix metalloproteinases and increased recruitment of immune cells that induces cervical remodeling that leads to PTB in human and animal models (Denison et al., 2000; Kuon et al., 2010; Kirby et al., 2016). While these findings suggest that progesterone supplementation maybe a useful therapeutic intervention for PTB prevention at least three large clinical trials have now demonstrated that vaginal progesterone does not significantly reduce preterm birth rates and in 2 of the studies, it did not reduce the rates of PPROM in subgroup analyses (O’Brien et al., 2007; Norman et al., 2016; Crowther et al., 2017; Norman and Bennett, 2017). The most recent PROLONG trial also demonstrated that another progestin 17αhydroxyprogesterone acetate did not significantly reduce recurrent spontaneous PTB (Blackwell et al., 2020). This has prompted some researchers to opine that it is now time to examine alternative therapies to progesterone for PTB prevention (Norman and Bennett, 2017). However, given the multiple mechanisms that may lead to PTB, research now needs to be focused on identifying the patient populations that may derive benefit from progesterone therapy.

Our study has several limitations. Firstly, we were unable to quantify MMP1 protein levels in part because the samples had to be diluted for IL8 level quantification using the magnetic Luminex assay. Another limitation of the study is that we only investigated inflammation induced molecular pathways, so our findings may not apply to other initiators of PPROM such as thrombin. Thrombin also induces *MMP1* mRNA expression, IL1β and IL8 protein levels in human amnion mesenchymal cells (Chigusa et al., 2016). In amnion mesenchymal cells this inflammatory response to thrombin can be inhibited by activators of nuclear factor erythroid 2- related factor 2 (NRF2) a transcription factor that mediates the expression of cell defense and antioxidant genes (Chigusa et al., 2016). Recently it has been demonstrated that GR signaling may also modulate NRF2 transcriptional activity, potentially highlighting the central role GR may play in pathways leading to PPROM and PTB (Alam et al., 2017).

In summary our findings provide additional evidence that the progestin MPA exerts its anti-inflammatory effects on molecular pathways implicated in PPROM through GR and not through PGRMC1 in fetal membranes. Identifying the downstream mechanisms by which GR exerts these effects could provide new insights into therapeutic interventions for PPROM prevention in at risk patients with the use of selective GR agonists and modulators.

## Data Availability Statement

The datasets generated for this study are available on request to the corresponding author.

## Ethics Statement

Ethical review and approval was not required for the study on human participants in accordance with the local legislation and institutional requirements. Written informed consent for participation was not required for this study in accordance with the national legislation and the institutional requirements.

## Author Contributions

WM performed experiments, data analysis and interpretation, drafting of the manuscript, and approval of the final version of the manuscript. LF contributed to the study concept and design, drafting of the manuscript, and approval of the final version of the manuscript. TA contributed to the study concept and design, performed experiments, data analysis and interpretation, drafting of the manuscript, and approval of the final version of the manuscript. All authors contributed to the article and approved the submitted version.

## Conflict of Interest

The authors declare that the research was conducted in the absence of any commercial or financial relationships that could be construed as a potential conflict of interest.
